# Nrf2 Down-Regulation by Camptothecin Favors Inhibiting Invasion, Metastasis and Angiogenesis in Hepatocellular Carcinoma

**DOI:** 10.3389/fonc.2021.661157

**Published:** 2021-06-09

**Authors:** Qian Liu, Shanshan Zhao, Fanguang Meng, Hankang Wang, Liwei Sun, Guijie Li, Feng Gao, Feng Chen

**Affiliations:** ^1^ Department of Radiology, The First Affiliated Hospital of Shandong First Medical University & Shandong Provincial Qianfoshan Hospital, Jinan, China; ^2^ Shandong First Medical University & Shandong Academy of Medical Sciences, Jinan, China; ^3^ Key Laboratory for Experimental Teratology of the Ministry of Education and Biomedical Isotope Research Center, School of Basic Medical Sciences, Shandong University, Jinan, China

**Keywords:** hepatocellular carcinoma, metastasis, reactive oxygen species, Nrf2, camptothecin

## Abstract

Higher oxidant stress capacity could promote invasion and metastasis. A previous study showed hepatocellular carcinoma (HCC) expressed more Nrf2 than para-carcinoma tissue. The chemotherapeutics such as epirubicin (EPI) could increase Nrf2 expression, while Camptothecin (CPT) could inhibit tumor growth by down-regulating the key molecule of antioxidant stress signal—Nrf2. The role of Nrf2 in invasion and metastasis was still unclear. In this study, we use EPI and CPT to determine the invasion and metastasis in Huh7 cells, H22 and Huh7 mouse models. In Huh7 cells, Nrf2 expression and ROS level were found increased after incubation with EPI by western blot and flow cytometry assay. But with the combination of EPI and CPT, inhibition of Nrf2 could decrease proliferation, invasion, and metastasis, which were investigated by CCK8 assay, wound healing, and Transwell assays. In Huh7 and H22 mouse models, EPI promoted Nrf2 up-regulation and nucleus translocation. Tumor growth was obviously inhibited with a single application of EPI or CPT. The combination of EPI and CPT could inhibit Nrf2 expression but demonstrated more suppressing effect of tumor growth than EPI. Western blot and immunohistochemical staining study revealed that Nrf2 inhibition was beneficial in decreasing the expression of N-cadherin, MMP9, Snail as well as Twist, and increasing E-cadherin, which were associated with epithelial–mesenchymal transition (EMT). Nrf2 down-regulation promoted lung metastasis of H22 cells *in vivo*. In addition, H&E staining and immunofluorescence staining of VEGFR suggested angiogenesis of Huh7 and H22 tumors was reduced. In conclusion, down-regulation of Nrf2 demonstrated inhibition of invasion, metastasis, and angiogenesis of hepatoma, which may provide a potential therapy in HCC.

## Introduction

Hepatocellular carcinoma (HCC) is the most common primary liver cancer and the fifth most common malignant cancer in the world (1, 2). Patients with HCC usually undergo intrahepatic or extrahepatic recurrence, accounting for about 90% of HCC related deaths (3, 4). Recently, oxidative stress, which elevated reactive oxygen species (ROS) production and promoted DNA damage and protein expression change, has been proved to be the main reason of the HCC development (5).

To maintain oxidative homeostasis, the tumor cells would activate nuclear factor E2-related factor 2 (Nrf2), the principal antioxidant response transcription factor. The Nrf2 pathway was the major regulator of antioxidant and cytoprotecting pathway (6). Under physiological conditions, Nrf2 is usually kept at the basal level in the cytoplasm and combined with Kelch-like ECH-associated protein 1 (Keap1) (7). Under oxidative stress, Nrf2 was detached from Keap1 after sensing the increase of ROS and then translocated to the nucleus, where Nrf2 dimerized with small MAF (sMAF) proteins, leading to a variety of downstream changes on anti-oxidant gene expression, such as NQO-1 and HO-1 (8–12).

Growing evidences suggested that over-expression of Nrf2 played an important role in the malignant cells’ migration, which was the major contributor to the mortality of cancer patients (13–15). Cancer cells utilized the epithelial–mesenchymal transition (EMT) program to execute the invasion and metastatic cascade (16). The undergoing of EMT process was demonstrated by down-regulating the expression of E-cadherin (the epithelial marker) and N-cadherin (the mesenchymal marker), following the increased expression of Snail and Twist (core EMT programs’ transcription factors) (17, 18). Meanwhile, the matrix metalloproteinase 9 (MMP9) contributed to invasion and metastasis through EMT in HCC (19). In the stage of tumor development, Nrf2 could promote the EMT by down-regulation of E-cadherin expression (20).

Angiogenesis was vital to tumor growth as it supplied the oxygen and nutrients (21). The ROS level affected the vasculature in a dose-dependent manner. Therefore, activated Nrf2 pathway could enhance angiogenesis *via* regulating the HIF-1*α*/VEGF axis (22, 23). Knockdown of Nrf2 resulted in a decrease of vascular endothelial growth factor (VEGF), the growth factor with important pro-angiogenic activity (24). Down-regulation of VEGF inhibited blood vessel formation and subsequently slowed down the tumor growth (25).

These evidences indicated that the activation of Nrf2 may be positively correlated with the invasion, metastasis, and angiogenesis of HCC. In our previous study, we demonstrated that the inhibition of Nrf2 by Camptothecin (CPT) suppressed the proliferation of HepG2 and SMMC-7721 cells by enhancing the cytotoxicity of chemotherapeutic drugs in HCC cells (26). Here, we would demonstrate the effect of Nrf2 down-regulation on invasion, metastasis, and angiogenesis of HCC *in vivo* and *in vitro* by single administration of epirubicin (EPI, a common chemotherapeutic drug) and combined administration of EPI and CPT, aiming to provide a novel strategy for HCC treatment.

## Materials and Methods

### Chemicals, Reagents, and Equipment

The ROS kit was purchased from Beyotime (Shanghai, China). Nrf2 antibody and MMP9 antibody were obtained from Proteintech (Wuhan, China). The E-cadherin, N-cadherin, Snail and VEGFR antibody were obtained from Cell Signaling Technology (MA, USA). The Twist antibody, *ß*-Tubulin and *ß*-actin antibodies were obtained from Bioworld (Illinois, USA). DMEM medium (High Glucose), RPMI 1640 medium, and fetal bovine serum (FBS) were provided by Biological Industries (Kibbutz Beit Haemek, Israel). Penicillin (100 U/ml) and streptomycin (100 µg/ml), 0.25% Trypsin Digestion solutions (without EDTA and phenol red), puromycin, and PIPA buffer kit with PMSF were obtained from Solarbio (China, Beijing). GLUTAMAX and Sodium Pyruvate were purchased from Gibco (South Australia, Australia). PBS buffer, TBST buffer, Crystal violet staining solution, H&E staining and immunofluorescence (IF) staining reagents were received from Servicebio (Wuhan, China). SDS-PAGE Gel Preparation Kit, protein maker, HRP-labeled Goat Anti-Rat IgG were provided by EpiZyme (Shanghai, China). ECL substrate was obtained from Merck Millipore (Darmstadt, Germany). CCK8 kit was provided by DOJINDO (Kumamoto Ken, Japan). Twenty four-well Transwell units and Matrigel were purchased from Coring Life Science (MA, USA). The epirubicin (EPI) and Camptothecin (CPT) were obtained from Selleck (Shanghai, China). The flow cytometry was performed in the CytoFlex counter of Beckman Company (CA, USA). The membrane of western blot was scanned by Tanon 4600 (Shanghai, China).

### Cell Culture

Huh7 (Human Hepatocellular carcinoma cell lines) cells were obtained from the China National collection of Authenticated Cell Cultures. H22 (Murine Hepatocellular carcinoma cell lines) cell lines were obtained from Shanghai Zhong Qiao Xin Zhou Biotechnology. The Huh7 and H22 cells were cultured in 37°C and 5% CO_2_. Huh7 cells were cultured in the DMEM medium (High Glucose) containing 10% fetal bovine serum (FBS), 1% GLUTAMAX, 1% Sodium Pyruvate, and 1% 100 U/ml penicillin, and 100 µg/ml streptomycin. H22 cells were cultured in the RPMI 1640 medium with 10% FBS, and 1% 100 U/ml penicillin and 100 µg/ml streptomycin.

### Generation of Nrf2 Knockdown Huh7 Cells

Huh7 cells (4 × 10^4^/500 μl) were seeded in 24-well plates and incubated at 37°C and 5% CO_2_, overnight. The HitransG A (20 μl) was added into the well for 12 h. Then, 10 μl Scramble negative control virus (sc RNAi) or lentivirus-shRNA Nrf2 virus (Nrf2-KD) (MOI = 20) was added into the well. After 16 h, the medium was removed, and the cells were covered with 2 ml DMEM complete medium for 48 h. Next, 2 ml DMEM complete medium with puromycin (4 μg/ml) was added into the well for 48 h. The transfection efficiency was observed in fluorescence microscope. The negative control virus or lentivirus-shRNA Nrf2 virus was purchased from Genechem (Shanghai, China). The targeted sequence was caGAGAAAGAATTGCCTGTAA.

### RT-PCR

Huh 7 cells (1 × 10^6^) were collected to the Ep tube, and the mRNA was extracted by TRIzol method. Briefly, 1 ml TRIzol was added to lyse the cells. Then 200 μl chloroform was added into the Ep tube and centrifuged. The upper transparent liquid was collected to the new tube and mixed with equal isopropanol. The tube was centrifuged, and the supernatant was discarded. Then 1 ml 80% ethanol (dissolved in DEPC water) was added into the tube. Next, the 80% ethanol was removed by centrifugation. The RNA was dissolved in 20 μl RNase free water. The mRNA concentration was measured by spectrophotometer. Then, inverse transcription was performed according to the description in Evo M-MLV RT-PCR Kit (AgBio, Hunan, China). The PCR was performed according to the description in Accurate Taq PCR Kit (AgBio, Hunan, China). The agarose gel electrophoresis was used to detect the expression of Nrf2 and internal control (GAPDH). NFE2L2 Primer: Forward: 5′-TCAGCGACGGAAAGAGTATGA-3′, Reverse: 5′-CCACTGGTTTCTGACTGGATGT-3′. GAPDH Primer: Forward: 5′-AAGGTGAAGGTCGGAGTCAAC-3′, Reverse: 5′-TGTAGACCATGTAGTTGAGGTCA.

### Animal Models

All animal experiments were performed according to the ARRIVE guidelines approved by the Animal Care and Use Committee of Shandong First Medical University. Male athymic nude mice and male BALB/c mice (6 weeks old) were purchased from Vital River Laboratory Animal Technology (Beijing, China) and fed under Specific pathogen free condition with free access to water and standard food.

In subcutaneous xenotransplant tumor models, 1 × 10^6^/200 μl PBS of Huh7 cells and H22 cells was implanted subcutaneously into male nude mice and BALB/c mice, respectively. When the volume of tumors was approximately 1.0–1.5 mm^3^, the mice were divided into four groups including the control group (PBS), the EPI group (EPI: 1 mg/kg), the CPT (CPT: 3 mg/kg) and combined groups (EPI combination with CPT injection). The mice models were administered with drugs through intraperitoneal injection every three days. The tumor volume was calculated on 0 day (first injection), 2, 4, 6, 8, 10 days with the equation: Tumor volume (mm^3^) = π/6 × Length × Width^2^ (27). After 10 days post first injection, the tumors were isolated and weighted. The tumor volume and weight were analyzed by GraphPad Prism and used to represent the tumor growth.

In lung metastasis mice model, 1 × 10^6^/200 μl PBS of H22 cells was injected to BALB/c mice *via* tail vein. 6 days later, the mice group were divided into the control group, EPI group, CPT group, and combined group following the treatment of PBS, EPI (1 mg/kg), CPT (3 mg/kg), and combination of two drugs every three days. After 10 days of drug administration, the lungs of mice were separated and weighted.

### ROS Level Detection

The Huh7 cells was adjusted to 1 × 10^6^/well in 2 ml complete DMEM medium and placed in six-well plates overnight. Cells were treated with 0.12 μM EPI (dilution in sterility PBS buffer) and 0.5 μM CPT (dilution in DMSO) for 48 h. After removing the medium, 10 μM DCFH-DA solution in serum-free DMEM medium (1 ml) was added into the well and incubated for 20 min. The cells were washed three times with serum-free DMEM medium to remove DCFH-DA. Then, cells were collected and resuspended in 0.5 ml PBS for analysis by flow cytometry.

### Western Blot

The tumors were smashed in the RIPA buffer with 1 mM PMSF on ice. The cells were treated for 48 h and were lysed. Next, tissue and cells were centrifuged, and supernatants were treated with loading buffer. The samples were loaded into the SDS-PAGE gel, and electrophoresis of transmembrane was performed. The target membranes were covered with blocking buffer and then incubated with appropriate antibody overnight. Then, the membranes were incubated with HRP-labeled Goat Anti-Rabbit IgG solution and HRP-labeled Goat Anti-mouse IgG solution. Finally, membranes were covered with ECL substrate and scanned. The protein expression was represented by the gray assay of targeted membrane.

### Immunohistochemical Staining and Immunofluorescence Staining

After 10 days post first injection, paraffin sections of tumors were deparaffinized and rehydrated. Then, the sections were incubated with EDTA antigen repair buffer (pH 9.0) to perform the antigen retrieval. Next, sections were blocked with BSA for 30 min and covered with appropriate antibody at 4°C, overnight. Next, the sections were treated with different secondary antibodies in IHC staining and IF staining. In IHC staining, the sections were incubated with HRP-conjugated secondary antibody, and then were added with DAB substrate. The hematoxylin was used to perform the nucleus counterstaining. Finally, the cell nucleus was blue, and the positive expression of the targeted protein was brownish yellow. In IF staining, the sections were covered with Cy3(red)- or FITC (green)-conjugated secondary antibody. DAPI reagent (blue) was added to sections to indicate the cell nucleus. The positive expression of targeted protein was green or red. The assay of protein expression in the tissue was analyzed by Image Pro Plus and represented as IOD/Area value.

### Cell Proliferation

The Huh7 cells were adjusted to 7,500 cells/100 µl of complete DMEM medium and added into 96-well plates overnight. Then, EPI and CPT were dissolved in 100 µl complete DMEM medium and treated to the cells. The final concentrations of EPI and CPT were 0.12 µM and 0.5 µM, respectively. Cell counting kit-8 (CCK-8) reagent (5 µl) was added to the cell suspension and OD value in 490 nm was measured in a microplate reader. Experiments were repeated four times.

### H&E Staining

The tumor, lung, and liver on 10 days post first injection were prepared in paraffin sections, and then stained following the specification of H&E staining kit. Briefly, following deparaffinization and rehydration, hematoxylin was added to the sections for 5 min. Then 1% acid ethanol reagent was covered on sections for 5 s. Then, the blue returning liquid promoted the tissue to return blue, and then the eosin solution was incubated with sections for 10 min. Finally, the sections were dehydrated and fixed with neutral balsam. The image was obtained under the optical microscope. The nuclear was blue, and cytoplasm was pink.

### Wound Healing Assays

Cells (1 × 10^6^/well) were added into six-well plates and cultured in complete DMEM medium overnight. Cells were scratched by a sterile pipette tip and washed with PBS for three times and cultured in DMEM medium without FBS. The cells were treated with 0.12 µM EPI and 0.5 µM CPT, respectively. The wounds were examined at 0, 24, and 48 h post scratch.

### Transwell Assay

Transwell assays for migration were performed in Transwell inserts of 24-wells with 8 μm pore polycarbonate membrane. The upper compartments were added with Matrigel (100 μl, 300 μg/ml). Huh7 cells were suspended in serum-free DMEM at 2.5 × 10^4^/ml. Then, the inner chamber was added with 200 μl cells, and the outer chamber was added with 750 μl complete DMEM medium. After 48 h, cells in the inner chamber were carefully removed. The migrating cells on the outside of the membrane were stained with Crystal violet solution buffer, following photographed under bright-field microscope (400× magnification). Each experiment was performed in triplicate.

### Statistical Assay

All experiments were performed at least three times and analyzed by GraphPad Prism. The data were expressed as mean ± SD, and one-way ANOVA with Tukey’s multiple comparison tests was used for analysis in two-group datasets at a significance level of P <0.05.

## Results

### CPT Down-Regulated ROS and Inhibited the Nrf2 Expression in HCC

**Figure 1:**
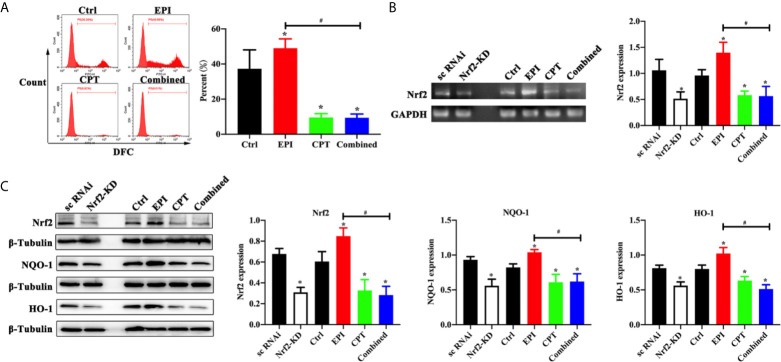
Expression of ROS level and Nrf2 expression with EPI and CPT administration in Huh7 cells. The non-transfected Huh7 cells were treated with EPI and CPT for 48 h. The sc RNAi and Nrf2-KD were transfected with scramble negative control and Nrf2-knockdown RNAi. **(A)** ROS level detected by flow cytometry after EPI and CPT intervention in Huh7 cells. **(B)** The mRNA expression and assay of Nrf2 by western blot in Huh7 cells. **(C)** The protein expression of Nrf2, NQO-1, and HO-1 in Huh7 cells. ^*^P < 0.05, ^#^P < 0.05.

EPI and CPT were used to treat the HCC cells so as to investigate the effect of ROS level in HCC cells. In **Figure 1A**, we found the ROS level in the EPI group was higher than that in the control group (P < 0.05). After treatment with CPT, whether in the CPT group or the combined group, ROS level was significantly decreased compared with that in the control group (CPT group *vs* control group, P < 0.05; combined group *vs* control group, P < 0.05). More importantly, the ROS level was lower in the combined group than in the EPI group (combined group *vs* EPI group, P < 0.05).

We generated the Nrf2 knockdown (Nrf2-KD) Huh7 cells, so as to analyze the effect of EPI and CPT on Nrf2 regulation in Huh7 cells. As shown in **Figures 1B, C**, we found Nrf2 expression was significantly decreased in the Nrf2-KD group compared with the scramble negative control group (sc RNAi) (P < 0.05). Nrf2 expression was higher in the EPI group than that in the control group. Nrf2 expression was significantly inhibited in the CPT group compared with that in the control group (P < 0.05). Meanwhile, Nrf2-KD significantly inhibited the expression of downstream proteins (NQO-1, HO-1) of Nrf2 (P < 0.05). In the EPI group, the expression of NQO-1 and HO-1 was higher than that in the control group (P < 0.05). In the CPT and combined groups, the expression of NQO-1 and HO-1 was significantly decreased in comparison with that in the control group (P < 0.05). Moreover, the combined group also showed lower NQO-1 and HO-1 expression than the EPI group. The level of Nrf2, NQO-1, and HO-1 in the CPT group and the combined group approximated that in the Nrf2-KD group. These results indicated the CPT inhibited the expression of Nrf2 and downstream protein (NQO-1 and HO-1) in Huh7 cells.

**Figure 2:**
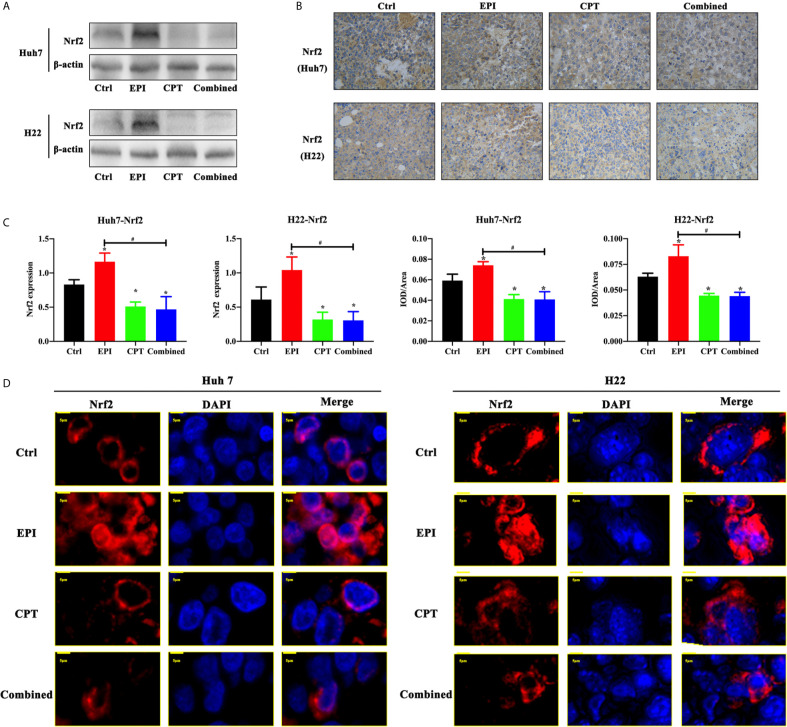
Expression of Nrf2 expression and translocation with EPI and CPT administration in Huh7/H22 tumor cells. The subcutaneous tumors of Huh7 and H22 were separated on 10 days post first injection of EPI and CPT. **(A)** The Nrf2 expression by western blot. *β*-actin was the internal control. **(B)** The Nrf2 expression and assay by IHC staining. **(C)** The assay of Nrf2 expression. **(D)** The nucleus translocation of Nrf2 (red) in Huh7 and H22 tumors. DAPI (blue) indicated the nucleus. ^*^P < 0.05, ^#^P < 0.05.

In order to detect the effect of EPI and CPT on Nrf2 regulation *in vivo*, western blot and IHC staining were performed in subcutaneous xenograft mouse models of Huh7 and H22 cells. In **Figures 2A–C**, we observed that treatment of CPT could down-regulate the expression Nrf2 compared with the control group (CPT group *vs* control group, P < 0.05). Although Nrf2 expression was obviously increased in the EPI group (P < 0.05), the combined administration of CPT with EPI could decrease the Nrf2 expression compared with the control group (P < 0.05) and the EPI group (P < 0.05). In the control group and CPT group, Nrf2 (red) was mainly distributed in the cytoplasm. Nrf2 translocated from the cytoplasm to the nucleus after EPI treatment, while Nrf2 translocation was found decreased after CPT treatment. These results indicated that CPT can inhibit Nrf2 nuclear translocation and affect its transcription function (**Figure 2D**).

### CPT Suppressed HCC Cell Proliferation and Tumor Growth by Nrf2 Down-Regulation

**Figure 3:**
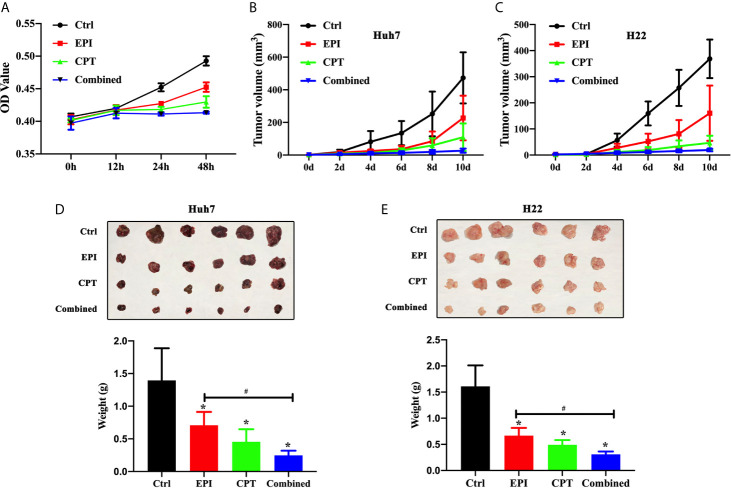
Down-regulation Nrf2 by CPT suppressed proliferation of Huh7 cells and tumor growth of Huh7 and H22. **(A)** Proliferation of Huh7 cells was measured and presented as OD value at 0, 12, 24, and 48h. **(B, C)** Tumor volume of Huh7 cells **(B)** and H22 cells **(C)**. **(D, E)** The tumor weight of Huh7 cells **(D)** and H22 cells **(E)**. ^*^P < 0.05, ^#^P < 0.05.

We investigated the effects of Nrf2 expression on the proliferation of Huh7 cells, as well as the tumor growth in subcutaneous xenograft Huh7 and H22 mice model. The results in **Figure 3A** suggested that the proliferation of Huh7 cells in the EPI, CPT, and combined groups was significantly lower than that in the control group at 24 and 48 h post drug treatment. The proliferation of Huh7 cells in the combined group was significantly slower than that in EPI group.

In Huh7 and H22 tumor bearing mice, the volume of tumors (**Figures 3B, C** **)** was measured on 2, 4, 6, 8, and 10 days and also weighed on day 10 (**Figures 3D, E** **)**, after drug treatment. The smaller tumor size and lower tumor weight were found in the EPI, CPT, and combined groups than those in control group. Suppression of Nrf2 by CPT in the combined group remarkably decreased the tumor size and the tumor volume compared with EPI groups.

### CPT-Induced Nrf2 Suppression Contributed to Inhibiting EMT Process

Molecular alterations of EMT progress after modulating Nrf2 expression were studied by IHC staining and western blot in Huh7 cells and Huh7/H22 tumor model treated with EPI and CPT.

**Figure 4:**
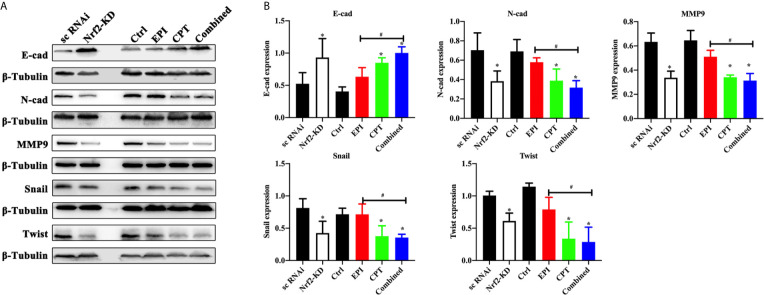
CPT suppressed EMT progress of Huh7 cells. The non-transfected Huh7 cells were treated with EPI and CPT for 48 h. The sc RNAi and Nrf2-KD were transfected with scramble negative control and Nrf2-knockdown RNAi. **(A)** The expression of E-cadherin (E-cad), N-cadherin (N-cad), MMP9, Snail and Twist was measured by western blot. **(B)** Densitometry assay of the band in western blot. *ß-*Tubulin was the internal reference. ^*^P < 0.05, ^#^P < 0.05.

In Huh7 cell lines (**Figure 4**), the Nrf2 down-regulation by Nrf2 RNAi significantly increased the expression of E-cadherin and decreased the expression of N-cadherin, MMP9, Snail and Twist, and MMP9. In the Nrf2-KD group, the CPT group and combined group, the expression of E-cadherin was increased and the expression of N-cadherin, MMP9, Snail and Twist was decreased compared with the control group. EPI had a significant effect on EMT process. These results demonstrated that Nrf2 down-regulation favored the inhibition of the EMT process.

**Figure 5:**
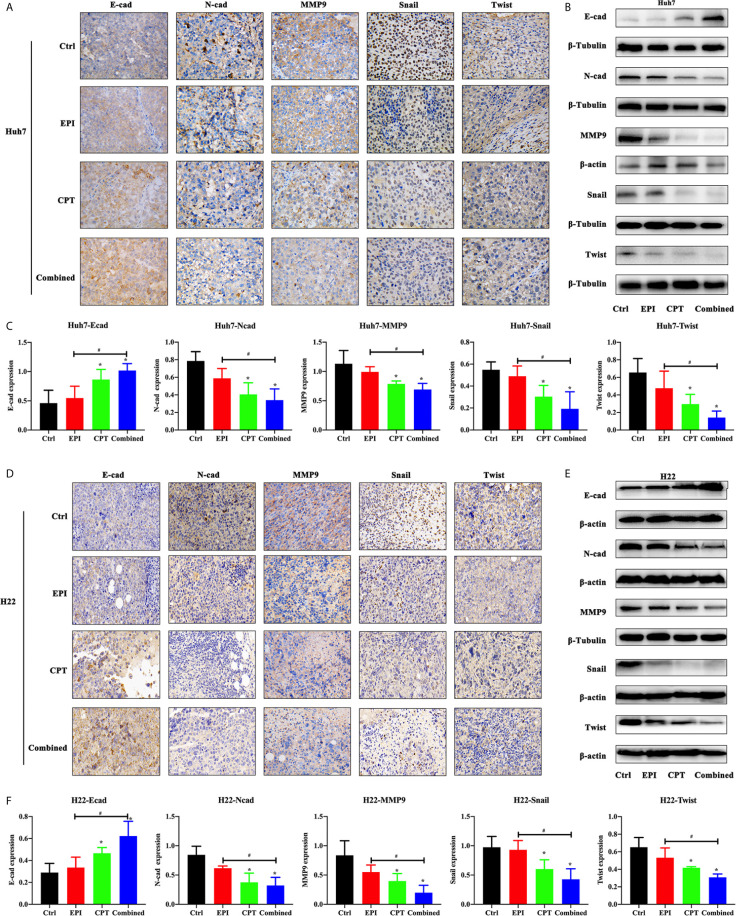
CPT suppressed EMT progress of subcutaneous xenotransplanted Huh7 and H22 tumors. The EMT progress assay was performed by subcutaneous xenotransplanted Huh7 and H22 tumors. **(A, B)** The expression of E-cadherin (E-cad), N-cadherin (N-cad), MMP9, Snail and Twist was measured by immunohistochemical staining **(A)**, and western blot **(B)** in Huh7 tumor. **(C)** Densitometry assay of the band in western blot in Huh7 tumor. **(D–F)** The expression of E-cadherin (E-cad), N-cadherin (N-cad), MMP9, Snail and Twist was measured by immunohistochemical staining **(D)**, and western blot **(E)** in H22 tumor. **(F)** Densitometry assay of the band in western blot in H22 tumor. *ß*-actin and *ß*-Tubulin were the internal reference. ^*^P < 0.05, ^#^P < 0.05.

In the Huh7/H22 tumor model (**Figure 5**), the expression of N-cadherin, Snail, Twist, and MMP9 was significantly decreased after CPT administration (CPT group and combined group), compared with the control group. The combined group had less expression of N-cadherin, Snail, Twist, and MMP9 than the EPI group. Expression of E-cadherin in the CPT and combined groups was higher than that in the control group. The western blot study further confirmed the finding in IHC staining.

### CPT Suppressed Invasion and Metastasis of HCC *via* Down-Regulation Nrf2

The wound healing and Transwell assays with Huh7 cells *in vitro* and lung metastasis study in H22 mouse model were performed so as to investigate the effects of Nrf2 expression on invasion and metastasis in HCC.

**Figure 6:**
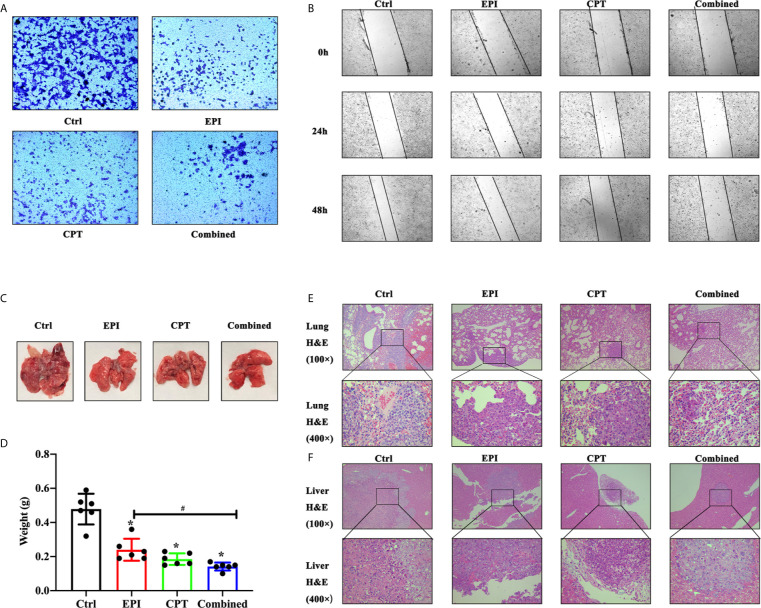
The invasion and metastasis assay of HCC *in vitro* and *in vivo*. **(A, B)** The invasion and metastasis assay of Huh7 cells *in vitro*. **(A)** Transwell assay showed the migration of huh7 cells at 48 h. **(B)** In wound healing assay, migration of Huh7 cells was examined at 0, 24, and 48 h. **(C–F)** The invasion and metastasis assays of H22 cells *in vivo* was detected by lung metastasis assay 10 days after the first drug injection. **(C)** The image of lung in mice model. **(D)** The assay of lung weight in mice model. **(E, F)** The H&E staining of lung **(E)** and liver **(F)**. ^*^P < 0.05, ^#^P < 0.05.

In the Transwell assay (**Figure 6A**), the numbers of migrated cells in the EPI, CPT, and combined groups were much fewer than that in the control group at 48 h post drug administration. And, the combined group showed less cell migration than the EPI groups.

In wound healing assay (**Figure 6B**), the wound closure in EPI, CPT, and combined groups was much narrower than that in the control group at 24 and 48 h. However, the wound closure in the EPI group was wider than that in the combined group after Nrf2 down-regulation.

In lung metastasis assay, the lung was weighted on day 16 post injection of H22 cells. The H&E staining of the lung and liver was also performed. In **Figure 6C**, great amounts of macroscopic H22 cell nodules were visible in the lung tissue in the control group, and more infiltration of H22 cells were observed by H&E staining in **Figure 6E**. After treatment with EPI and CPT, the macroscopic H22 cells’ nodules decreased, and lung weight was remarkably reduced (**Figure 6E**). Lung weight was found lighter in the combined group than that in the EPI group (**Figure 6D**). More interestingly, the H22 cells nodules were also found in liver tissues (**Figure 6F**). The size of tumor was smaller in the EPI, CPT, and combined groups compared with that in the control group. Some parameters in the combined group, such as the amount of tumor nodules, lung weight, H22 cell infiltration, and the size of tumor metastasis in the liver, were much poorer than those in the EPI group.

### CPT Inhibited Angiogenesis of HCC *via* Down-Regulation Nrf2

The expression of angiogenesis in tumor tissues was analyzed in Huh7 and H22 tumor bearing mice by H&E and IF staining of VEGFR after treatment with EPI and CPT.

**Figure 7:**
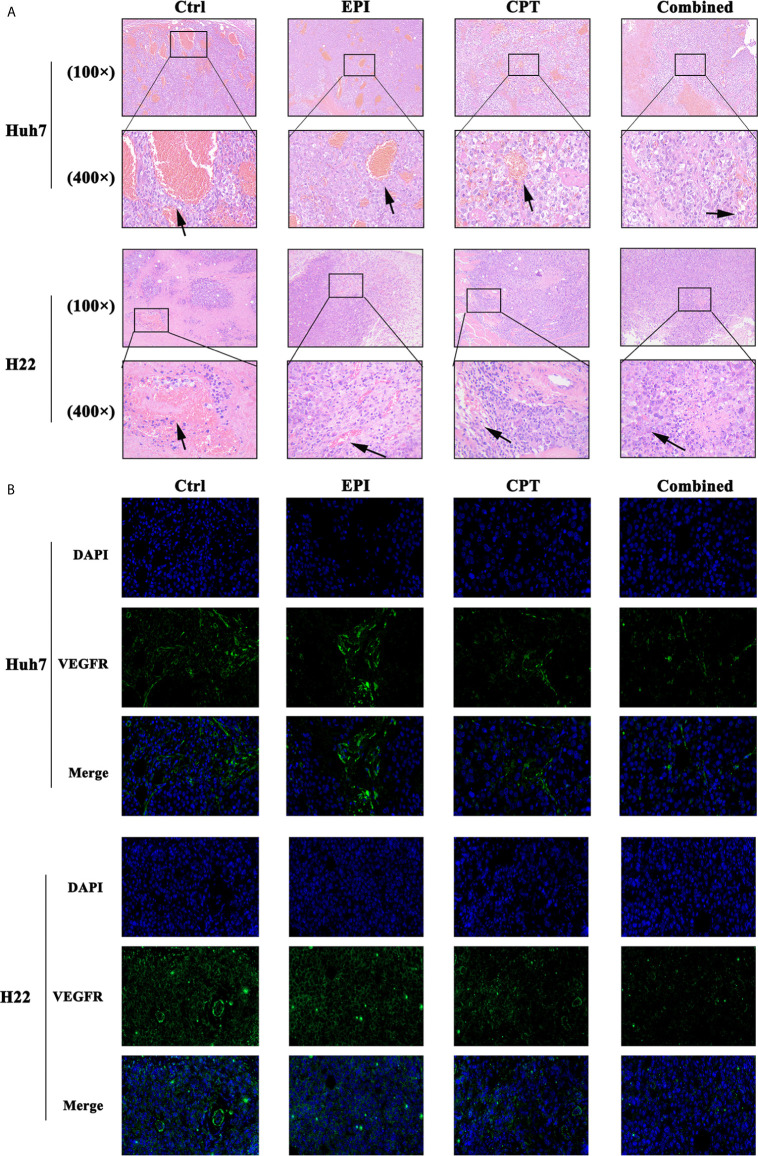
CPT administration favors suppression of angiogenesis. **(A)** The H&E staining of the Huh7 and H22 tumors at 100× and 400× magnification. The arrow showed the vessel inside the tumor. **(B)** The IF staining of VEGFR (green) in Huh7 and H22 tumor at 400× magnification. DAPI (blue) indicated the nucleus of the tumor cells.

H&E staining of Huh7 and H22 tumors (**Figure 7A**) indicated that more vessels were found in the control group compared with the EPI group. Interestingly, vessels in the control group were thicker than that in the EPI group. After Nrf2 down-regulation by CPT intervention, the number and the thickness of vessels were obviously reduced in comparison with the control group. Moreover, the combined group also exhibited fewer vessels than the EPI group, and the vessels in the combined group were found finer.

In addition, angiogenesis in the EPI group was less than that in the control group (**Figure 7B**). As Nrf2 was down-regulated by CPT administration, the combined group demonstrated lower VEGFR expression than the control group. Meanwhile, lower VEGFR expression was found in the combined group than that in the EPI group.

## Discussion

Cancer cells exhibit fast metabolism, which requires a high concentration of ROS to maintain their fast proliferation (28). High level of ROS could lead to nucleus genomic instability to accelerate the progress of the cancer development, including promoting cell proliferation, invasion, and metastasis by activating plenty of signal proteins (29–31). ROS in cancer cells could also be increased by different therapeutic strategies, such as chemotherapy, radiotherapy, and other methods (32, 33). In tumor chemotherapy, the chemotherapeutic drugs could enhance the killing efficiency of tumor *via* inducing intracellular ROS response. However, increased ROS level could also induce abnormal activation of Nrf2 and then promote the migration and metastasis of tumor (19, 34). Therefore, inhibiting the activation of Nrf2 pathway in the tumor was vital to inhibit the progression of tumor. A large number of studies have proved that Nrf2 stayed in lower activation state in normal tissues. In contrast, primary tumors and metastatic tumors showed high Nrf2 expression and Nrf2 activation (22, 23). Our previous study showed that HCC had more Nrf2 expression than para-carcinoma tissue, and the high expression of Nrf2 promoted the progress of HCC and its chemotherapy resistance (24). Therefore, targeting Nrf2 maybe provide a novel strategy to suppress the tumor progression.

As a common chemotherapeutic drug for HCC, EPI has the characteristics of general chemotherapeutic drugs (35). In the metabolic process, EPI could kill the tumor by inducing ROS accumulation, promoting the overload of intracellular calcium and inducing apoptosis and necrosis of tumor (29, 30). However, the high ROS level induced by EPI may result in abnormal activation of Nrf2. Abnormal activation of Nrf2 enhanced tumor invasion and metastasis of tumor through cascade reaction of downstream proteins. Now, many evidences have confirmed that Nrf2 activation could inhibit the degradation of Bach1, and then promote the progress of lung cancer (23, 31).

Nrf2 was the main regulator against ROS response in cells, and it could control the antioxidant response elements (AREs) by interaction with small MAF proteins (36). Nrf2 was the central protein of the Kelch-like ECH-associated protein 1 (Keap1)-Nrf2-ARE pathway (37). In the classical pathway, Keapl protein mediated the ubiquitination degradation of Nrf2 protein when ROS was in physiological level. However, when ROS was increased, Nrf2 which was no longer degraded by Keap1 could enter the nucleus to activate the downstream antioxidant genes (38, 39). Then Nrf2 translocated and regulated the antioxidant proteins to protect cells against oxidative stress *via* controlling the various oxidation resistance genes, such as glutamate cysteine ligase (GCL), glutathione reductase (GSR), SLC7A11 (40–42). In our previous study, we have screened out an effective Nrf2 inhibitor—camptothecin (CPT)—from thousands of clinical drugs by ARE luciferase reporter assay (20, 24). CPT could specifically inhibit the activity of Nrf2 without increasing the level of intracellular ROS (26). As a long-standing clinical chemotherapeutic drug, its safety has been fully confirmed (43, 44). Moreover, the dosage of Nrf2 inhibitor in our study (*in vivo*: mg/kg) was lower than that for chemotherapy in clinics (8 mg/kg) (26). It was also proved that combination of CPT with EPI could effectively inhibit Nrf2 expression in HepG2 and SMMC-7721 cells (26). Considering that tumor progression was mainly affected by tumor proliferation, invasion, metastasis, and the amount of angiogenesis, the Nrf2 inhibition by CPT may provide a novel exploration about invasion, metastasis, and angiogenesis in HCC treatment. In this study, we aimed to prove that CPT do not increase the risk of HCC progression as the conventional chemotherapeutical drug, and to provide a new strategy for clinical control of HCC progression.

CPT was a chemotherapeutic drug used in clinics for a long time, and many studies suggested that CPT could inhibit the expression of Nrf2 (45, 46). In our study, CPT was also proved to effectively inhibit ROS level and Nrf2 expression, while EPI could increase ROS level and induce high Nrf2 expression. Additionally, co-administration of CPT could suppress ROS generation and Nrf2 expression induced by EPI.

Nrf2 participate in cell proliferation by various pathways. Nrf2 knockout cells proliferated more slowly than wild-type cells (47–49). Over-expression of Nrf2 could modulate mRNA translation to promote tumor cell proliferation (50). Fan reported that Nrf2 promoted the proliferation of glioma cells through specifically adjusting ROS level and inducing resistance to ferroptosis (51). Our results indicated that EPI, CPT, and combination of EPI and CPT inhibited cell proliferation and tumor growth of HCC. However, EPI treatment induced Nrf2 expression and inhibited the proliferation of Huh7 cells as well as the tumor growth of Huh7 and H22. It might be due to that EPI could induce other inhibition patterns of cell growth, such as pro-apoptosis, G2/M arrest by CDK1 down-regulation, as well as the limitation of the nutrition supply towards cancer cells (52–54). After down-regulating the EPI-induced Nrf2 by CPT, the proliferation and tumor growth of HCC were obviously decreased. All these strongly suggested that down-regulation of Nrf2 by CPT favored the inhibition of HCC growth.

EMT was the major biological process for tumor cell migration and invasion (55). In this process, cancer cells would lose connection with the basement membrane and get a capability of migration and invasion, following increasing degradation of extracellular matrix (ECM) (56). The core regulatory of EMT promoting transcription factors including E-cadherin, N-cadherin, Twist and Snail participated in this process (57). Down-regulation of E-cadherin cannot maintain normal epithelial cell morphology, resulting in the destroyed adhesion system and then inducing the tumor from non-infiltration to infiltration. Transcription factor Snail and Twist could directly or indirectly inhibit the activity of E-cadherin to promote EMT (58). N-cadherin played an important role in neovascularization and adhesion between tumor cells and mesenchymal cells. In esophageal squamous cell carcinoma cells and human ovarian cancer cells, Nrf2 promoted EMT phenomenon by down-regulation of E-cadherin and up-regulation of N-cadherin through some unclear mechanisms (31, 59, 60). Matrix metalloproteinase, such as MMP9, was required in the EMT process of cancer cells *via* degrading the cellular adhesion (61, 62). In Huh7 knockdown cells, the EMT process was inhibited. Although EPI could inhibit HCC growth, it did not suppress the EMT process. After Nrf2 down-regulation by CPT, the EMT process was also inhibited. These results demonstrated that Nrf2 down-regulation favored inhibition of the EMT process.

It was also reported that some cancer cells with strong metastatic capacity exhibited high Nrf2 expression (63). Therefore, the wound healing, Transwell assays, and lung metastasis were performed to detect the invasion and metastasis ability of HCC after treatment with CPT. *In vitro* and *in vivo* studies demonstrated that EPI could inhibit invasion and metastasis in a certain level. This may be due to the inhibition of cell proliferation by EPI, resulting in slower invasion and metastasis. After inhibition of the Nrf2 expression by CPT, the cell invasion and metastasis were suppressed. Meanwhile, our previous study indicated Nrf2 knockdown Huh7 cells had slower invasion and metastasis. These results revealed that Nrf2 down-regulation by CPT benefited for the suppression of invasion and metastasis.

The high metabolism of tumor in the growth process could result in a hypoxic micro-environment in the interior tumor. At this stage, the tumor could activate the growth factor such as VEGF and induce vasculature generation (64). Then VEGF activated VEGFR and promoted endothelial cell proliferation as well as tubule formation (65). In some xenograft models, knockdown of Nrf2 could reduce vasculature formation (66, 67). In HCC tumors, vascular quantity in different groups was found in the order: control group >, EPI group >, CPT group >, combination group. The difference of tumor volume and weight in different groups may be due to the different degrees of vascularization. In addition, there was difference in sensitivity to CPT between Huh 7 tumor and H22 tumor, probably due to the difference of angiogenesis. Furthermore, CPT treatment could also inhibit the expression of VEGFR in the tumor. All these illustrated that CPT could suppress Nrf2 and inhibit angiogenesis of HCC.

Although Nrf2 inhibition by CPT could suppress the progress of HCC, several limitations of the present study should be considered. The binding site of CPT on Nrf2. It would be interesting to explore the mechanism of Nrf2 inhibition.

## Conclusion

In this study, CPT could inhibit the up-regulation of ROS and Nrf2 expression which were induced by EPI treatment. After down-regulation of Nrf2, Huh7 and H22 cells exhibited slow growth, weak invasion and metastasis, together with decreased angiogenesis. Therefore, Nrf2 may be a potential target for prediction of HCC metastasis.

## Data Availability Statement

The original contributions presented in the study are included in the article/supplementary material. Further inquiries can be directed to the corresponding authors.

## Ethics Statement

All animal experiments were performed in agreement with the ARRIVE guidelines. The protocol was approved by the Animal Care and Use Committee of Shandong University with the corresponding ethical approval code (LL-201602040, 2016-2022).

## Author Contributions

FC and QL designed the studies. QL and SZ carried out the study, including data collection and data analysis. FM, HW and LS performed data analysis. QL wrote the original draft. SZ and FG edited the manuscript. GL and FC supervised. All authors contributed to the article and approved the submitted version.

## Funding

This study was funded by the Nature Science Foundation of China (grant no. 81803008 to FC) and the Natural Science Foundation of Shandong Province (grant no. ZR2019BH041 to FC).

## Conflict of Interest

The authors declare that the research was conducted in the absence of any commercial or financial relationships that could be construed as a potential conflict of interest.
